# Design, implementation and evaluation of a web-based computer-tailored intervention to prevent binge drinking in adolescents: study protocol

**DOI:** 10.1186/s12889-018-5346-4

**Published:** 2018-04-04

**Authors:** Marta Lima-Serrano, José Manuel Martínez-Montilla, Joaquín S. Lima-Rodríguez, Liesbeth Mercken, Hein de Vries

**Affiliations:** 1 Department of Nursing, School of Nursing, Physiotherapy and Podiatry, University of Seville, Seville, Spain; 2 Department of Health Promotion, Maastricht University, School for Public Health and Primary Care CAPHRI, Maastricht, The Netherlands

**Keywords:** Adolescence, Alcohol drinking, Binge drinking, I-change model, Web-based interventions, Computer-tailoring

## Abstract

**Background:**

Binge-drinking in adolescents is a highly prevalent healthcare problem that associates physical and mental health complications with community implications. This paper describes the design, implementation and evaluation of the first web-based computer tailored intervention aimed at the prevention of binge drinking in Spanish adolescents.

**Methods:**

The *Alerta Alcohol* program is based on the I-Change Model. First, feedback from focus and Delphi groups are used for cultural adaptation and to obtain further information on the items to be included on the program. A pilot study is then conducted to assess feasibility and to identify strengths and weaknesses. Second, a Cluster Randomized Controlled Trial is conducted to test the effectiveness of *Alerta Alcohol* in students aged 16 to 18 years. The study is performed in 16 high schools from Andalusia (southern Spain), which are randomized either to the experimental or the control condition (EC and CC). The EC receives the *Alerta Alcohol* intervention, which consists of four sessions at school (baseline questionnaire, two sessions in three scenarios: at home, celebrations, and public places, and a final evaluation). The adolescents are provided with answers related to their views of each scenario; this information is used to provide highly specific feedback regarding their knowledge, risk perception, self-esteem, attitude, social influence, and self-efficacy. In addition, two booster sessions are given at home to reinforce the previous messages. The CC just completes the baseline and the final evaluation questionnaires and then they are allowed to receive the intervention as well (as a waiting list). Evaluation takes place after four months. The primary endpoint is binge drinking within 30 days prior to the evaluation and alcohol use in the previous week. It is expected that *Alerta Alcohol* reduce the prevalence of binge drinking by 10%. Follow up analyses are carried out to determine the differences in effectiveness according to the compliance of the program (quality of the implementation).

**Discussion:**

The results are expected to be applicable and may incorporate improvements in the practice of the Healthcare and Education Systems. If the program proves to be effective, regional and eventual national implementation should be considered.

**Trial registration:**

Trial registration number (ClinicalTrials.gov): NCT03288896. This study was retrospectively registered on 19/09/2017.

## Background

The consumption of alcoholic beverages is a public healthcare problem worldwide, causing 3.3 million deaths every year [1, 2]. The toxicity associated with excessive alcohol ingestion and its enormous addictive power makes alcohol one of the most consumed and dangerous drugs [1]. Moreover, alcohol is linked to several conditions like brain damage, cardiovascular lesions, cancer, suicide, sexually transmitted diseases, alcohol dependence and premature death. Alcohol consumption also affects society, leading to high financial and social burden, as in cases of violence, crime, accidents, etc. [3].

Alcohol drinking has become endemic, and part of the social and cultural life, resulting in a great social permissiveness and a low perception of its risks. It is widely available, easy accessible and lacks restrictive legislation [4]. In Spain, traditional drinking patterns are classically associated to the adult population, characterized by daily drinking, usually during meals, although hardly ever leading to acute intoxication. However, in the recent years, important changes have occurred concerning the amount of ingested alcohol and the drinking patterns, particularly in the adolescent and the young adult population [5]. Binge drinking, defined as consuming five or more drinks by men, or four or more by women, within a short period of time [6, 7], is increasingly prevalent among youngsters aged 15 to 24, in whom it is becoming the usual pattern of consumption.

Regarding Spanish adolescents, the national survey ESTUDES (2016) showed that the prevalence of binge drinking at the age of 16 was 37.1%, which doubled that of 14-year-olds (14.2%), and reached 44.2% at the age of 17 [8]. These figures highlight the importance of preventing binge drinking.

**Fig. 1:**
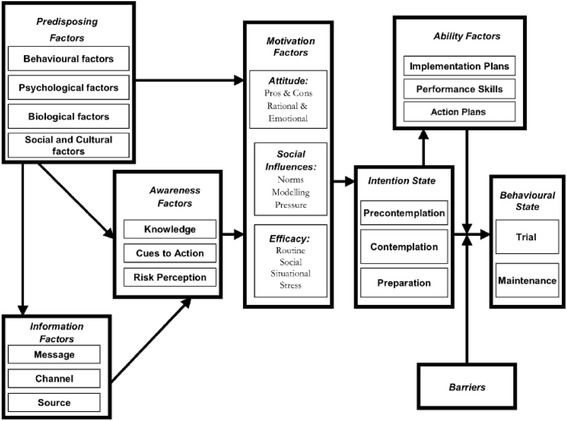
I-Change model. (Figure obtained from De Vries, Lezwijn, et al., [12])

For predicting healthy behavior acquisition, several theoretic models, borrowed from health and social psychology, have been used [9]. The present study is based on the I-Change Model that integrates elements of various social cognition and self-regulation models, as well as principles from socio-ecological models [10–12]. It has been used before to study alcohol dependency and seems to influence alcohol consumption (Fig. 1) [13–17]. The I-change model states that a behavior is the result of an individual’s intentions, action plans and abilities. The intention of an individual can range from no intention at all for change (pre-contemplation) to a real intention of changing a certain behavior (preparation). The individual’s abilities and environmental constraints determine whether the intentions will be accomplished [18, 19]. In addition, the rejection of consumption seems to be influenced by different individual skills [4], like the ability to carry out action plans that may increase the chances of going from intention to action.

Specifically, the I-Change Model focuses on three motivational factors: the person’s attitude (pros and cons) towards a certain behavior, the social influence experienced (modelling, norms and pressure), and his or her self-efficacy expectations, which influences the intention of a person to develop a specific behaviour [10–13, 18, 20–22].

Regarding alcohol abuse prevention among adolescents, most of the programs are carried out at schools, for they are considered the most suitable places for learning interventions and where children and adolescents stay for significant amounts of time [23–25]. In Spain, previous national preventive programs of alcohol abuse in adolescents have been conducted in the school environment [4, 26]. The use of Internet based programs at schools is growing, specifically those concerning the prevention of alcohol abuse [22, 27, 28]. Of particular interest is the utilization of computer tailored technology (CTT), which provides highly personalized behavioral and motivational feedback to the individual. On one hand, the Internet has become a world wide available and promising tool, accessible to large populations [29]. On the other hand, a previous meta-analysis has shown that computer tailoring (CT) is effective in supporting health-related changes for a number of different behaviors [30]. Moreover, for the adolescent population, Internet based programs may also be a motivational and attractive tool to work with [27, 31].

CTT is a method of assessing individuals’ behaviors and opinions and selecting communication content on the basis of their answers, using data-driven decision rules that produce personalized feedback automatically from a database of content elements [30]. CT has shown advantages, such as accessibility, ease in filling out the questionnaires in the appropriate time, the possibility of obtaining tailored advice at the participant’s request, and it has shown to be cost-effective [32]. In addition, CT consists of dynamically tailored interventions (assessing intervention variables prior to each feedback), opposed to static tailoring (providing one baseline assessment on which to base all successive feedbacks), that has shown to increase efficacy over time [30].

Similar previous studies, aiming to evaluate web-based healthcare programs in adults have been conducted [14, 33–35]. Computer tailoring has been used to promote healthy food and physical activity in adolescents [32] and in the prevention of drug dependence, such as smoking [36] or excessive alcohol drinking [27, 37, 38]. In this regard, Jander et al. [27, 28] developed a dynamically tailored internet based intervention in the context of a tailored game, for the prevention of excessive alcohol drinking in Dutch adolescents, aiming to change the motivational factors and the actual occurrence of binge drinking. In this intervention, also based on the I-change model, the adolescents received personalized information on their consumption behavior, as well as messages aimed at its prevention. It resulted effective in binge drinking prevention among 15 and 16-year-old participants beginning to consume alcohol, although efficacy was not confirmed in older groups.

The goal of this paper is to describe the design, implementation and evaluation of the first web-based computer tailored intervention aimed at the prevention of binge drinking in Spanish adolescents (*Alerta Alcohol*). This program is a dynamically web-based computer-tailored intervention, which is delivered at schools and is an adaptation of the Dutch program [27]. First, we designed a web-based computer-tailored intervention, given that certain cultural differences may exist between the Dutch and the Spanish adolescent population. In this regard, feedback from focus groups with adolescents and parents were analyzed to consider their viewpoints regarding the risk and protective factors of binge drinking. The information obtained from these interviews was used for the development of the CT intervention, which was later assessed by a Delphi Panel and by a pilot study. Second, a two-arm Cluster Randomized Controlled Trial (CRCT) aimed at testing the effectiveness of the *Alerta Alcohol* program is then performed.

## Methods

### Intervention design

**Fig. 2:**
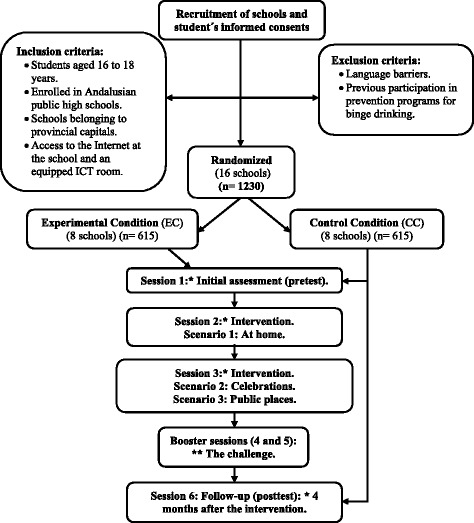
Flowchart of the intervention. * It takes place in the school environment. ** It takes place in the participant’s home

A CRTC was designed, with one experimental and one waiting-list control condition randomized at the school level (EC and CC), with an initial (pre-test) evaluation and a final (post-test) evaluation, performed four months after the intervention (Fig. 2). To design the program, we followed the study by Jander et al. [27, 28], which was carried out in the Netherlands with an equivalent objective. The CONSORT guidelines were followed [39]. The independent variable was the participation versus non-participation of the student in the *Alerta Alcohol* program.

### Participants

The study was conducted with students aged 16 to 18 years belonging to the public-school system. In order to calculate the sample size, the online GRANMO tool was used (http://www.imim.cat/ofertadeserveis/software-public/granmo/). According to the ESTUDES 2016 study, in Spain, the prevalence of adolescent binge drinking within the previous 30 days is 32,2% [8]. It is estimated that the intervention reduces the consumption by 10%. Accepting a significant *p* value < .05, a statistical power of .80, for a two-sided test, 615 subjects were required for the CC and 615 for the EC (1230 participants) to find a statistically significant proportion difference, expected to be of .32 for CC and. 22 for EC. Following the study by Jander et al., it has been anticipated a drop out rate of about 50%. The ARCSINE approximation was used [27].

### Selection of the sample

Collaboration was requested from the Educational Plans and Programs Service of the Department of Education, Culture and Sports of the Government of Andalusia. A total of 16 high school centers, two from each province of Andalusia (southern Spain) were contacted and randomly assigned to either the EC or the CC. The participating students in the CC were considered to be on a waiting list, and were allowed to receive the intervention voluntarily once the study was finalized. For the selection of centers, the criteria were: 1. Public secondary education schools from Andalusia; 2. Belonging to provincial capitals; 3. Access to the Internet at school and an equipped ICT room available for the student. From the list of centers that met these criteria, a random selection was made.

Participation in the study was confirmed by e-mail, telephone or, when necessary, by visit. A formal letter and an informative folder was sent to each center. If they accepted the collaboration, the inclusion criteria were checked.

All students enrolled gave consent to participate and had an Internet access point in their homes. Those with language difficulties or those who had previously participated in prevention programs of binge drinking were excluded.

The selected schools were not blinded, since the EC needs to schedule a total of four sessions during school hours. In the first session, the reference questionnaire (pretest) is filled out throughout January/February. For the second and third sessions, computerized interventions were carried out. There was a 1–2-week period between sessions. Then, in a fourth session at school, the follow-up questionnaire (post-test) was carried out throughout May/June. Each session takes approximately one hour.

The CC only gets two sessions: baseline data in January/February 2017, and the follow-up questionnaire (posttest) four months later, in May/June 2017.

### Intervention

#### Design of the web-based computer-tailored intervention

**Fig. 3:**
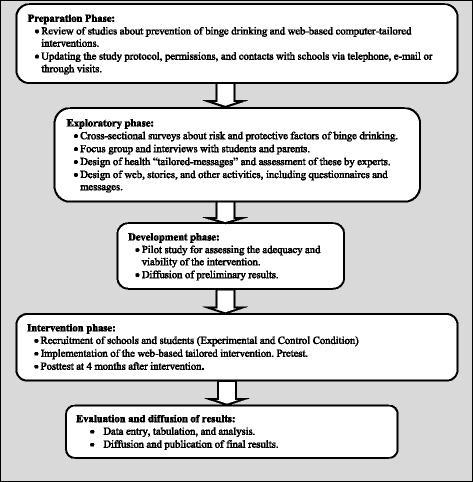
ALERTA ALCOHOL: study timeline

The design is developed using several strategies (Fig. 3):

1. Because this study is based on the Dutch program, it is reasonable that certain cultural differences may exist. Consequently, the first phase of the project consists of gathering 10 to 14 focus groups, each with 6–8 adolescents 16 to 18 years old, as well as, focus groups with fathers/mothers [13]. The goal of these studies is to identify in detail the patterns of alcohol consumption, binge-drinking, binge drinking intention and the cognitive and motivational variables determining factors such as attitudes and self-efficacy. The group interviews are recorded and analyzed by members of the research team, resulting in a list of the most important items to be addressed in the Spanish computer tailoring intervention.
2. In a second phase, the feedback from these interviews is used for the development of the computer tailored intervention *Alerta Alcohol*. We designed tailored health messages about the needs of adolescents, which were assessed by Delphi expert groups. To make the program more attractive, we designed stories with avatars (based on the focus group interviews). The group of experts also reviewed and consulted the content of these stories.
3. The third phase consists of piloting the Program with a sample of 100 students. The appropriateness and the feasibility were analyzed. Using scales like the Likert five-point scale, we assessed: the name of the program, joint evaluation of the web-based computer-tailored activities, extension, perceived interest, credibility, acceptability, understanding, ease of use and perceived impact.

Feedback from the participants in the pilot study was used for the final design of the program used in the CRCT, which was carried out subsequently [40].

#### Program content

The *Alerta Alcohol* program consists of preventive messages and information about the benefits of not consuming alcohol, reducing the positive attitudes and encouraging the negative attitudes towards alcohol drinking and binge drinking, as well as social influence and self-efficacy, as personalized feedback. Skills and action plans are encouraged to help the student to reject binge drinking. This information is presented as different tailored messages, personalized using the student’s first name and gender. Moreover, taking into account the results from the focus groups, we designed the program using terminology adapted to the age and gender of the participant. Finally, we designed four avatars or characters (two males and two females) that can be chosen by the participant as part of the story development.

The *Alerta Alcohol* program consists of a short story in which the main character wakes up after an evening in which he/she consumed alcohol excessively, and does not remember what happened. There are two different stories based on the participant gender: one for boys and one for girls (however, very similar). The stories take place in three different scenarios (at home, at celebrations and in public places). Under these scenarios, the story is presented and questions and tailored messages are offered. We used strategies to reinforce certain behaviors. For example, we developed different messages customized with the names of the participants to provide personalization and using elements like repetition of the answer, showing respect and empathy, counter persuasion, introducing social modeling and new beliefs, and reinforcing the positive behaviors and motivational feedback [41, 42].

**Fig. 4:**
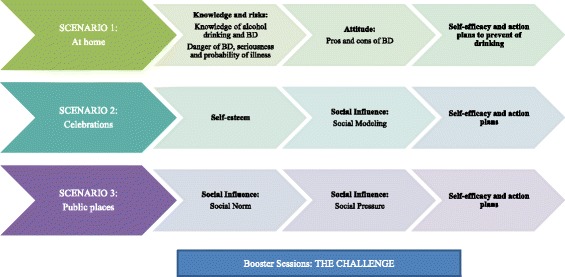
Description of the intervention routings

The questions and tailored messages are related to alcohol drinking and binge drinking and based on the I-change model and its central concepts (attitude, social influences, self-efficacy and action planning) (Fig. 4). For all scenarios, self-efficacy is reinforced and specific action plans are offered to the adolescent in order to reject alcohol and binge drinking in situations that incite to such behaviors. In addition, we developed questions and tailored messages aiming to increase self-esteem and awareness of factors such as the acknowledgement of risk perception of alcohol drinking and binge drinking.

To start the intervention, students are provided with access to a website. This website has a section in which they can create an account to get access to the intervention. In this account, they select their school, which determines their control or experimental status.

The intervention consists of six sessions. A baseline questionnaire followed by three different scenarios (sessions 1–3), a fourth session in which adolescents may accept the challenge of not consuming alcohol in excess in an upcoming event, a session to evaluate the challenge (session 5), and session 6 which is the evaluation of the intervention or post-test evaluation. The control condition only receives the pre-test and post-test (sessions 1 and 6), whereas the experimental condition performs all the sessions.

##### Session 1 or baseline

Participants begin responding a baseline questionnaire (with a series of measurement variables), including demographics, alcohol use behaviors, and mediator variables such us risk perception, motivational determinants (attitude, modeling, social influences, self-efficacy) and intention to decrease alcohol consumption.

##### Session 2

In this session, participants are confronted with the first scenario (at home), in which the knowledge and risk of alcohol drinking and binge drinking are addressed, as well as the degree of danger and seriousness of binge-drinking, probability of a medical complication, and pros and cons of binge drinking. The feedback is focused on providing information on the general and individual consequences of alcohol drinking and binge drinking, as well as making the adolescent aware of the negative aspects of binge drinking and reinforcing the negative beliefs about it with the aim of changing to an attitude against alcohol abuse.

##### Session 3

In this session, participants continue with the second and third scenarios. In the second scenario (celebrations such as Christmas, wedding parties or festivals), healthy messages include how to manage self-esteem, providing information on its importance. In addition, the scenario addresses issues related to social models, helping the adolescents to choose models considered most appropriate, encouraging them to seek support from friends and family members who do not drink alcohol in excess.

The third scenario (a public place) addresses issues related to social norms regarding binge drinking, like the opinion of others. It helps adolescents to deal with the perceived approval of drinking among family and friends, and to choose true relationships that may help them to avoid alcohol drinking and binge drinking. In addition, questions and messages also address how to resist the social pressure to drink alcohol, coming from friends or family members.

In all the scenarios, a last step is included, in which self-efficacy towards handling these situations is assessed. The answers are used to develop specific suggestions on how to cope with these situations, and specific action plans are offered to adolescents to handle alcohol and binge drinking under these scenarios. Adolescents are provided with a list of action plans for each scenario and are invited to indicate which specific action plans they are likely to undertake.

##### Session 4

One week after the last scenario, an additional session is offered at home. At this point, “*The Challenge*” is proposed: not to consume alcohol in excess in an upcoming drinking event. Adolescents receive an email inviting them to respond to a question on their alcohol drinking during the previous week. Then, they receive feedback on their drinking behavior in comparison to baseline, in order to create awareness of their own consumption [27]. Afterwards, they are questioned whether they have an event in the next 30 days where they might consume alcohol excessively. If so, adolescents are dared to accept the challenge (“I challenge you NOT to drink alcohol in this event, or at least, not to drink four or more glasses if you are a girl or five glasses or more if you are a boy”). If they accept the challenge, the date of the event is registered, as well as the type of event (at home, celebration, public place). Then, they are invited to build their own action plan with the aim of supporting their attempt not to drink. If they are not willing to make their own plan, they are provided with a list of plans that they previously suggested (in sessions 2 and 3) that they would likely follow for the specific scenario of the drinking event, since action plans are important for behavior changing in adolescents [18, 27]. Besides, participants receive feedback from sessions 2 and 3 to booster their motivation (attitude, social norms, modeling, and pressure). Finally, one day before the drinking event takes place, an email is sent as a reminder of the acceptance of the challenge. This mail is meant to self-monitor their behavior at the drinking event.

##### Session 5

On the day after the drinking event, the adolescent is invited to respond to a brief questionnaire about it, and whether he achieved the goal of not consuming four/five glasses of alcohol. If the adolescent manages to achieve this goal, a congratulatory message is given to reinforce the positive behavior; if he did not manage to achieve it, he is asked about the reasons, so that he/she can receive feedback and information about external and internal reasons for that behavior and what to do about it. In this feedback, adolescents are encouraged to continue avoiding/reducing alcohol consumption by using a cue reminder (an object that helps them remember not to consume four/five glasses of alcohol) at the next social event [27, 43]. Finally, adolescents are asked to repeat the challenge if they wish to.

##### Session 6 or evaluation

Four months after the first session, all participants complete the evaluation questionnaire which includes the same measurement variables as the baseline questionnaire.

##### Invitations and reminders

The first session is performed in the presence of a member of the research team. When participants create the account, they have to provide their e-mail. This e-mail is used to send invitations and reminders to participate. The first invitation is sent to the participants of the experimental condition who have not completed sessions 2 or 3. On week after completing session 3, participants receive an invitation to “*The Challenge*” (session 4). They receive a reminder two days before the drinking event. Then, two days after the drinking event a new message is sent to the participants who accepted the challenge in order to evaluate it (session 5). One and two weeks after session 6, students who have not completed it receive a reminder to complete the post-test.

In addition, schools receive reminders (emails and phone calls) to complete sessions 2, 3 and 6 (which are supposed to be implemented at schools).

### Measurement instruments

We use a Spanish version of the self-administered online questionnaire that was designed for the Dutch youth population, which has been previously validated [27, 44].

#### Social-demographic variables

Gender (male/female), age, parents’ educational level (none, primary, compulsory high school, higher secondary/vocational, university). Social status is measured by the social affluence scale (Does your family have own car or van? Do you have your own room at home? During the last twelve months, how many times have you gone on holiday with your family? How many computers does your family have?). We also ask about whether they have a tablet or a smart-phone [45].

The family functional status is evaluated with the Family Apgar Test, broadly used to evaluate the functioning of families with an adolescent child. It consists of five questions answered by a Likert three-point scale, assessing the adaptability or resource mobilization (Are you satisfied with the help you received from your family when you have problems?), participation or cooperation (Do you talk at home about the problems that you have?), development or growth (Are important family decisions discussed together at home?), resolution or capacity of spending time with a family member (Are you satisfied with the time that you spend together with your family?), and affection (Do you feel that your family loves you?) [46].

#### Drinking behavior

We assess two drinking patterns: weekly drinking and binge-drinking in the previous 30 days.

To assess weekly drinking behavior, adolescents indicate whether they have been drinking alcohol within the previous 7 days and if they did, how many glasses of alcohol they drunk. Based on this information we calculate the total amount of alcohol taken in the previous week [27].

To assess binge drinking (e.g. having 4/5 or more glasses of alcohol in one occasion for a girl/boy), we asked adolescents how many binge-drinking occasions they had in the previous 30 days.

#### Risk perception

We explore the perception of danger related to binge drinking, as well as the seriousness of health problems related to binge-drinking (such as liver problems, alcoholism or traffic accidents), and the probability of acquiring these problems. We use Likert scales with five answer options (from never to almost always).

#### Attitude towards binge drinking

We explore four items measuring pros (e.g. “Drinking 4/5 or more glasses of alcohol helps to have fun with my friends”); and four items measuring cons (e.g. “I don't like myself when I drink 4/5 or more glasses of alcohol”). We use Likert scales with five answer options (1 = absolutely disagree; 5 = absolutely agree).

#### Social influence. Model, norms and social pressure

*Social modeling* is assessed by asking participants how often people in their environment (i. e. parents, siblings, (best) friend(s), girlfriend/boyfriend) drink alcohol and engage in binge drinking (1 = never; 4 = very often).

*Social norm* is measured for each person in their direct environment (i. e. parents, siblings, (best) friend(s), girlfriend/boyfriend) by one item “My (e.g. mother) thinks that” …1 = “I am certainly not allowed to drink 4/5 glasses or more of alcohol” to 5 = “I am certainly allowed to drink 4/5 glasses or more of alcohol”.

*Social pressure* is assessed by “Have you ever felt pressure from (i. e. parents, siblings, (best) friend(s), girlfriend/boyfriend) to drink 4/5 or more glasses of alcohol?” We use a five-point scale (1 = never; 5 = always).

#### Self-efficacy

Self-efficacy is measured by ten items. Each item assesses whether participants feel able not to drink in a certain difficult situation (situations that would usually trigger binge drinking, e.g. “How difficult or easy is it for you not to drink more than 3 (if you are female) or 4 (if you are male) glasses of alcohol if others around you drink 4/5 glasses or more of alcohol?”). We use a five-point scale (1 = very difficult; 5 = very easy).

#### Intention

We used two questions about the intention of alcohol use and binge drinking with a five-point scale (1 = absolutely will not; 5 = absolutely will), “Are you intending to generally reduce your drinking in one occasion (e.g. in a bar, at a party etc.)” and “Are you intending to drink less than 4/5 glasses of alcohol in one occasion (e.g. in a bar, at a party etc.)”

#### Process evaluation

To assess the implementation compliance, the number or sessions performed by the participants are registered. After completing each session and in the posttest, we ask respondents whether the intervention was useful, realistic, interesting, and personally relevant on a five-point Likert scale (e.g., 1 = Totally disagree; 5 = Totally agree).

In the final evaluation, we also assessed the general satisfaction (e.g., 1 = Very unsatisfied; 5 = Very satisfied) and, by using a five-point Likert scale, the opportunity of learning, and the probability of using the counseling (e.g., 1 = Totally disagree; 5 = Totally agree) [38].

### Procedures and ethics approval

The implementation of the intervention is carried out in a school context and the students are who fill out the questionnaires. The first session in the different centers is guided by a member of the project, who also provides access to the website and register on the *Alerta Alcohol* platform. Then, sessions 2 and 3 are conducted by the teacher at school, and sessions 4 and 5 are self-administered by adolescents at home. The final evaluation or session 6 is again conducted by the teacher at school. Before initiating the participation in the study, parental and individual informed consent must be obtained.

The interventions are carried out according to bioethical guidelines: the students need to answer the questionnaires themselves and confidentiality is guaranteed. Informed consent and an online form to collect data are used. The study has the approval of the Bioethical Committee of Andalusia. The registration number for clinical trial is: PI-0031–2014. Registration date: 04 August 2015.

### Data analyses

Descriptive analyses are performed to describe the characteristics of the participants. Subsequently, multivariate analyses by multiple linear regression are conducted to analyze the effect of the program on the weekly consumption of alcohol, and the intention of reducing alcohol drinking. A binary logistic regression aiming to assess the effect on binge drinking within the last 30 days is performed. The independent variable was participating vs. not participating in the program, and co-variables were the outcome at baseline, socio-demographic variables and explored risk factors, which showed significance after a previous bivariate analysis. Analyses are conducted to explore the differential effects of the socio-demographic characteristics like age, gender or social status. Cases of linearity, homoscedasticity, normality of the quantitative variables, independence of errors, and non-co-linearity are taken into account. Also, analyses are carried out to determine the differential effect, according to the program compliance (i.e., number of performed sessions by participant), on the program outcomes. This may eventually provide information about the influence of the quality of the implementation on the program outcomes and of a possible implementation threshold effect.

The analysis of the data is supported by the R statistical program. A level of significance of *p* < .05 is used, and the size of the effect is calculated (r coefficient and Odds Ratio), with 95% confidence intervals.

## Discussion

Alcohol drinking in adolescents is a socio-health problem of crucial importance, because of its high prevalence and the physical and mental health risks affecting the adolescent consumers, in addition to the psychosocial and community implications derived from alcohol abuse [3–6, 47]. This paper describes the design, implementation, and evaluation of the first CT tailored intervention aimed to prevent alcohol drinking and specifically binge drinking in Spanish adolescents aged 16 to 18.

An important issue regarding program implementation at schools is compliance according to the original scheme, since implementing school programs often requires cooperation from teachers, who may find it difficult to provide the intervention as it was originally conceived. Thus, delivering an online intervention, i.e. a web-based computer-tailored intervention, may improve the program implementation, specifically regarding its compliance, integrity, and replicability, so that objectives can be achieved [48].

The high drop out rate is an important problem in a web-based computer-tailored intervention [27, 28, 33, 49]. Jander et al. [28] obtained a very low adherence to the complete program (N total = 824; 31.1%), so that optimal achievements could not be obtained at the finalization of the intervention. According to that study, they could not confirm a global effect of the intervention over binge drinking behavior in Dutch adolescents. Nonetheless, the intervention was effective in reducing binge drinking in adolescents between 15 and 16 years that completed at least two sessions. Additionally, they showed that a longer use of this intervention was associated with stronger effects for binge drinking.

In order to minimize the drop out rate, we followed different strategies to improve the characteristics of the program. First, we designed the intervention considering the feedbacks from focus groups with adolescents and parents. Second, we developed a dynamic intervention, with different interactions and stories adapted to gender and age [30]. Third, a Delphi expert group gathered to assess the program questionnaire and the tailored messages. Fourth, we carried out most of the interventions at schools as part of the health promotion curriculum. Fifth, reminders on participation were also sent via emails when participants had not finish the intervention procedures, so they could complete them out of school. Finally, concise, direct and personalized relevant messages were sent to promote the adhesion to the intervention [25, 27, 28].

In the adaptation of the intervention, we think that some changes were needed for the *Alerta Alcohol* program. There are different cultural issues related to alcohol drinking among different countries. For instance, drinking behavior can be associated with certain cultural or seasonal events, such us “Spring Break” in the United States or certain folk festivals like the Oktoberfest in Germany. In Spain, there is an alcohol drinking phenomenon called “*Botellón*”, which is the most prevalent event for alcohol drinking in adolescent population and consists in drinking alcohol in public places [21]. In Spain, around 70% of adolescents between 16 and 18 years old have participated in “*Botellón*” in the last 12 months [8]. This may be partially attributed to the prohibition of selling alcohol in bars and pubs to individuals younger than 18 years of age. Therefore, we decided to replace the bar scenario by public places.

Furthermore, since previous studies have found that a large proportion of adolescents may be in a pre-motivational phase, we believe it is reasonable to focus on prior aspects, relatively easier to modify, like factors of awareness (knowledge, action clues and risk perception) [50]. In this regard, along with the motivational factors (attitude, social influences, self-efficacy and action plans), which are necessary to initiate and maintain a healthy behavior [11, 20–22], in our intervention we emphasize awareness factors, like knowledge about alcohol drinking or binge-drinking and risk perception. According to Prochaska et al. [51] becoming aware is the first step to promote a change in health behavior.

In addition, we believe that it is necessary to provide with messages aiming to improve self-esteem, since low self-esteem is considered a determinant for risky behaviors in adolescents, such as alcohol drinking or binge-drinking. This is particularly relevant in the adolescence period, because self-esteem is a critical factor affecting the psychological and social fitting [52, 53].

In summary, this study tests the effectiveness of an intervention focused on reducing alcohol drinking and specifically binge drinking in Spanish adolescents between 16 and 18 years of age. If the program proves to be effective, the ultimate goal would be regional and eventual national implementation.

## Abbreviations

CC: Control condition
CRCT: Cluster randomized controlled trial
CT: Computer tailoring
CTT: Computer tailored technology
EC: Experimental condition
ICT: Information and communication technology
